# Insights into Adenovirus Uncoating from Interactions with Integrins and Mediators of Host Immunity

**DOI:** 10.3390/v8120337

**Published:** 2016-12-21

**Authors:** Glen R. Nemerow, Phoebe L. Stewart

**Affiliations:** 1 Department of Immunology and Microbial Science the Scripps Research Institute, La Jolla, CA 92037, USA; 2 Department of Pharmacology and Cleveland Center for Membrane and Structural Biology, Case Western Reserve University, Cleveland, OH 44106, USA

**Keywords:** adenovirus, AFM, capsid disassembly, integrins, defensins, cryoelectron microscopy, antibody, neutralization, nuclear pore, dynein, hexon, penton base, protein VI, *ts1*, cysteine protease, nuclear porins, microtubules, adenovirus receptors

## Abstract

Human adenoviruses are large (150 MDa) nonenveloped double-stranded DNA (dsDNA) viruses that cause acute respiratory, gastrointestinal and ocular infections. Despite these disease associations, adenovirus has aided basic and clinical research efforts through studies of its association with cells and as a target of host antiviral responses. This review highlights the knowledge of adenovirus disassembly and nuclear transport gleaned from structural, biophysical and functional analyses of adenovirus interactions with soluble and membrane-associated host molecules.

## 1. Introduction

Viruses spend significant time and resources getting “dressed for battle” against the host in a process known as assembly. This event produces stable, mature particles that have the capacity to survive the potentially harsh environment prior to host cell invasion. However, capsid assembly is only half the battle in the world of viruses vs. host. A subsequent confrontation—cell invasion—requires “sequential disrobing” (disassembly) of key capsid proteins in the appropriate subcellular location at the right time. The organized manner of virus disassembly ensures the efficient delivery of the viral genome to the nucleus and may enable the virion to avoid detection by some components of the host immune system.

## 2. Uncoating (Disassembly) Is a Key Element of the Adenovirus Infection Cycle

Adenovirus disassembly promotes two events that are needed for later steps in cell entry. The first of these is the release of protein VI molecules located on the inside of the viral capsid [1,2,3,4,5,6]. Protein VI serves several important functions in the virus infectious cycle including acting as a cofactor for the viral cysteine protease [7,8], providing increased stability on the inner capsid shell [9], and most importantly, causing the disruption of the early endosome [5,6,10,11]. Disruption of the endosome allows partially disassembled virions to gain access to the cell microtubule transport system needed for capsid transport to the nucleus. A temperature sensitive mutant adenovirus designated *ts1* [12] fails to undergo capsid disassembly when grown at the non-permissive temperature and as a result, cannot release protein VI in the early endosome [6]. These mutant virions become trapped and degraded in endolysosomes [13] or recycled out of the cell [14].

Exposure of adenovirus to the endosomal environment also prepares the particle for transport to the nucleus [15]. Specifically, capsids undergo conformational changes during virion disassembly and this allows recognition of the altered particle by the dynein microtubule motor protein which facilitates virion transport to the nucleus [16]. Precisely what factor or factors trigger adenovirus conformational changes within the endosome is unknown. Constant-pH molecular dynamics simulations for alphaviruses and bacteriophage HK97 have identified specific residues in these viruses that respond to changes in pH and lead to a shifting of the stability between two conformational states [17,18]. In the case of adenovirus, it is possible that the low pH of the endosome triggers a conformational change, or alternatively perhaps ions or enzymes in high concentration in the endosome are responsible.

After reaching the nuclear pore complex, additional alterations in the virion capsid occur as a consequence of the actions of distinct microtubule motor proteins as well as capsid interactions with nuclear porins (Nups) [19]. This final uncoating step likely allows liberation of the viral nucleic acid from the capsid paving the way to nuclear import, although the precise trafficking events underlying this final step are currently poorly characterized. Thus, capsid disassembly is a complex process that brings about further changes in the virus particle needed for subsequent cell trafficking events that are crucial for cell infection.

## 3. Initial Interactions with Cell Receptors Trigger Capsid Disassembly

One of the first capsid proteins to dissociate from the virion is the fiber [4,20,21]. Fiber shedding at the cell surface was first demonstrated for human adenovirus type 2 (HAdV-C2) with anti-fiber and anti-hexon antibodies and co-precipitation experiments [4]. The cellular localization of fibers to the cell surface early during viral cell entry was confirmed by immunocytochemistry with adenovirus infected cells. In a later study, also using HAdV-C2, the cellular requirements for fiber shedding were investigated [20]. Integrins and filamentous actin were found to be essential for fiber release. This study also showed that although fiber shedding occurs early during viral cell entry, fiber release is not required for endocytosis. Fiber release and viral entry via endocytosis appear to be independent events during adenovirus cell entry. Quantitative live-cell fluorescence resonance energy transfer (FRET) assays with HAdV-C5 confirmed that the first significant change in adenovirus virions during cell entry is detachment of the fibers at the cell surface [21]. In the same study, fluorescent lifetime imaging microscopy (FLIM) indicated that the fast fiber shedding process occurs at the beginning of endocytosis. The capsid dissociation steps that transpire upon interaction with a host cell can be mimicked in vitro with heat treatment [6]. Mass spectrometry based studies of adenovirus disassembly triggered by mild heat treatment indicate complete release of fiber and penton base proteins, as well as partial release of other capsid proteins, after heating [22].

The fiber plays a critical role during cell entry in that it attaches to the primary receptor at the cell surface [23]. For most cell types and for most human adenovirus types, the attachment receptor is either a Coxsackie and Adenovirus receptor (CAR) [24] or CD46 (membrane cofactor protein) [25]. Other identified adenovirus attachment receptors include sialic acid-containing oligosaccharides, GD1a glycan, and Desmoglein-2 (DSG-2) [26,27,28]. Numerous crystal structures have elucidated the nature of the fiber/attachment receptor interaction [26,29,30,31,32,33,34,35]. These structures reveal that it is the distal fiber knob domain that interacts with the various cellular attachment receptors.

Integrins are essential for fiber release [20] and α_v_ integrins in particular serve as internalization receptors for adenovirus [36,37]. Many adenovirus types display an RGD (arg-gly-asp) loop at the top of the penton base protein and these RGD loops interact with a variety of integrins [28,37]. The penton base/integrin internalization receptor interaction triggers numerous signaling events [38,39,40], which lead to virus internalization via clathrin-mediated endocytosis and macropinocytosis. The interaction of adenovirus with two different cellular receptors, typically CAR and α_v_ integrin, via two different capsid proteins—fiber and penton base respectively—sets the stage for ripping the fiber from the virion and sets in motion the process of viral disassembly. This concept is supported by live cell microscopy studies indicating that virus binding to CAR gives rise to drifting motions of CAR on the cell surface while the engaged integrins remain immobile [41]. Specifically, the fiber/CAR interaction triggers actomyosin-2 dependent drifts, which generate pulling forces on the fiber. This pulling force on the fiber, together with the penton base/integrin interaction and resulting confinement of the penton base, results in disruptive forces that lead to breakage of the fiber/penton base connection. As Burckhardt et al. [41] have envisioned, either CAR pulling on the virus, or multiple CAR molecules pulling on the virus in different directions, would trigger the initial step of capsid disassembly. The adenovirus fiber is shed or released from the viral capsid, and remains associated with the cell surface, by virtue of the fact that the interaction between the fiber knob and the attachment receptor is a higher affinity interaction than that between the fiber and the penton base.

Structural studies have elucidated the nature of the interaction between the fiber and penton base protein within the adenovirus capsid [42,43,44]. This interaction involves a short conserved region at the N-terminal end of the fiber which sits at the interface between penton base monomers at the top of the penton base. The fiber/penton base interaction appears to be highly conserved among adenovirus types. Two notable points about the fiber/penton base interaction suggest that it is a weak point in the capsid structure and thus suitable for beginning the process of capsid disassembly. First, the interaction involves a relatively short peptide stretch within the fiber (F11 to Y17 in HAdV-C2 and HAdV-B35) [42,44]; secondly, the position of the fiber N-terminal interaction region between two monomers of the pentameric penton base suggests that its binding pocket may be vulnerable to disruption if the penton base undergoes a conformational change.

## 4. Adenovirus–Integrin Interactions May Destabilize the Capsid Vertex

Cryo-electron microscopy (cryoEM) studies have provided structural information on the interaction of integrin with the adenovirus penton base [45,46,47]. The adenovirus–integrin complex is challenging for structural biology as the integrin binding site on the capsid is on a highly flexible loop [42]. CryoEM has the advantage over X-ray crystallography in that ordered crystals are not needed for structure determination. However, like X-ray crystallography, cryoEM has difficulty resolving flexible regions of a protein complex. In general, a cryoEM single particle reconstruction of an ordered and symmetrical complex with a flexible region will show either no density or blurred density for the flexible region. The exception to this is if there is limited flexibility then it might be possible to sort particle images of defined states for the flexible region from the larger dataset. In this case, there is a possibility of reconstructing defined density for the flexible region in a particular state.

Before any adenovirus/integrin complexes were examined by cryoEM, an early study examined the adenovirus interaction with a monoclonal antibody, called DAV-1 [45]. DAV-1 is an anti-penton base antibody with reactivity against several different adenovirus types. Inhibition studies showed that either the full length monoclonal antibody (mAb) or the antigen-binding (Fab) fragments from DAV-1 blocked penton base binding to A549 cells, supporting the idea that DAV-1 binds to the integrin binding site on the penton base. Curiously, pre-incubation of cells with DAV-1 Fab fragments, but not DAV-1 mAb, blocked adenoviral infection. The immunoglobulin (Ig)G form of DAV-1 was found to have a higher affinity for penton base than Fab fragments. Therefore, affinity differences could not explain the observation of selective neutralization by Fab fragments but not intact IgG molecules.

Affinity-directed mass spectrometry was used to identify the epitope of the DAV-1 monoclonal antibody and the minimal epitope was found to be 9-residues in the RGD-containing loop of the penton base (IRGDTFATR). At the same time, a cryoEM structure of the complex of HAdV-C2 with the DAV-1 Fab fragment showed that DAV-1 binds to five protrusions on the top of the penton base. Therefore, the combination of mass spectrometry identification of the epitope, together with a moderate resolution (19 Å) cryoEM structure, resulted in localization of the integrin-binding RGD loop of the penton base. This occurred before the crystal structure of the penton base was determined [42] and well before atomic resolution structures of intact adenovirus were determined [2,3,48].

Positioning crystal structures of Fab fragments into the cryoEM density of the complex indicated that the penton base RGD loop must have considerable flexibility, which is presumably functionally significant for interaction with integrins. Modeling also indicated that steric hindrance from the fiber, combined with the presence of a few bound IgG molecules and the observed epitope mobility, might block binding of intact IgG molecules to all five protrusions on one penton base. This would leave a few RGD protrusions available for interaction with integrin, even in the presence of a few bound IgG molecules, and explain the observation that DAV-1 IgG molecules are not neutralizing. In contrast, five copies of the smaller Fab fragments could be modeled above the penton base protrusions, clarifying how a smaller Fab fragment might neutralize while the corresponding larger IgG molecule cannot.

The first cryoEM studies of adenovirus–integrin complexes were performed with both HAdV-C2 and HAdV-A12 and a soluble recombinant form of αvβ5 integrin [46]. HAdV-C2 has a long and highly flexible RGD loop containing ~79 amino acid (aa) residues. HAdV-A12 was chosen for a comparison structure because its integrin-binding RGD loop is significantly shorter (~17 aa). Both the HAdV-C2 and HAdV-A12 integrin complex structures revealed rings of integrin density above the penton base. However, the integrin density in the HAdV-C2 complex was more diffuse, presumably due to the longer and more flexible RGD loops. The resolutions of these structures were moderate, ~21 Å, but at this resolution it was possible to observe two discrete subdomains for the bound integrin. As part of the same study, kinetic analysis indicated ~4.2 integrin molecules bound per penton base at close to saturation, despite the fact that the penton base has five RGD binding sites for integrin.

**Figure 1:**
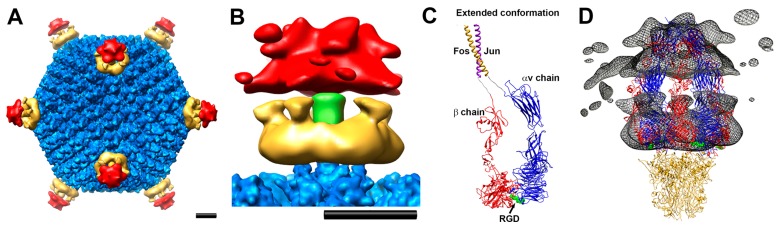
Cryo-electron microscopy (cryoEM) structural analysis of adenovirus–integrin interaction. (**A**) Full human adenovirus (HAdV)-A12/soluble-αvβ5-integrin structure viewed along a two-fold icosahedral axis. The icosahedral capsid is shown in blue, the proximal integrin density in gold, and the distal, more diffuse integrin density in red; (**B**) Enlarged side view of the vertex region with the fiber shaft in green. Scale bars, 100 Å; (**C**) Model of an extended conformation of the soluble form of αvβ5 integrin with Fos/Jun dimerization domains used for the cryoEM study. The bound RGD (arg-gly-asp) peptide is shown in green; (**D**) The cryoEM HAdV-A12/αvβ5 integrin density (mesh) docked with four copies of the integrin ectodomain in an extended conformation. Reprinted with permission from [47]. Copyright © American Society for Microbiology, Journal of Virology, 83, 2009, 11491–11051, doi:10.1128/JVI.01214-09.

**Figure 2:**
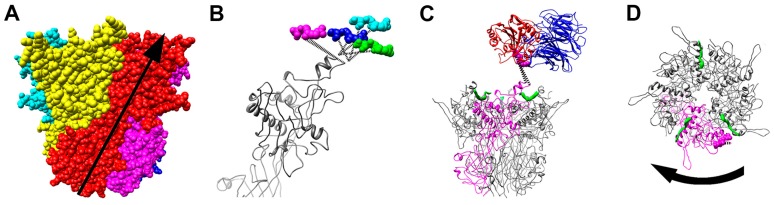
Natural twist of the penton base and possible untwisting by integrin. (**A**) Space-filling representation of the HAdV-C2 penton base pentamer (PDB-ID 1X9T) with each subunit in a different color. The natural twist of the penton base from the bottom of the pentamer to the solvent accessible surface in the virion is represented by an arrow; (**B**) Side view of four superimposed penton base monomers with four copies of the RGD residues (magenta, cyan, blue, and green) as modeled in the HAdV-A12/αvβ5-integrin cryoEM density. The dashed lines represent the extension of the flexible RGD loops. Note that the magenta copy of the RGD residues extends the RGD loop counter to the natural twist of the penton base subunit; (**C**) Side view of the penton base pentamer with one subunit in magenta, fiber N-terminal peptides in green, the magenta copy of the RGD residues as shown in panel B, and docked RGD-binding integrin domains (blue and red); (**D**) Top view of the penton base pentamer with a curved arrow indicating the direction of the possible untwisting precipitated by interactions with integrin. Reprinted with permission from [47]. Copyright © American Society for Microbiology, Journal of Virology, 83, 2009, 11491–11051, doi:10.1128/JVI.01214-09.

Ten years later, a higher resolution cryoEM structure was pursued for the HAdV-A12-αvβ5 integrin complex [47]. Subnanometer resolution (~8 Å) was achieved for the icosahedral capsid, while only ~27 Å was achieved for the integrin density due to the flexibility of the RGD binding site and incoherent averaging of bound integrin molecules (Figure 1). Docking the αvβ3 and αIIbβ3 integrin crystal structures into the cryoEM density indicated that a maximum of four integrins would fit above each penton base. The modeling also indicated that the close spacing of the RGD protrusions (~60 Å) on the penton base prevents four integrins from binding in identical orientations relative to the penton base. In other words, binding of four integrin molecules may cause one of the flexible RGD loops to be pulled in a direction that would lead to untwisting of the penton base pentamer (Figure 2). It was hypothesized that integrin binding may induce conformational changes in the penton base, which initiates further uncoating of the virus and release of membrane lytic protein VI molecules.

## 5. Mechanochemical Properties of the Adenovirus Capsid Provide Clues to Capsid Disassembly

Atomic force microscopy (AFM) has gained increasing use as a method to test the mechanical properties of virus capsids at the single particle level [49,50,51,52,53,54,55]. For AFM analyses, virions are first immobilized onto glass surfaces and the structure of the capsid is initially analyzed in the imaging mode using a ~20 nanometer (radius) probe connected to a cantilever. An associated laser records the topological features of the capsid as the cantilever probe rides over the surface of the virion. This process allows the orientation of the icosahedral virion to be determined via the location of the five-fold, two-fold or three-fold axes. The AFM probe is then switched to indentation mode and the mechanical forces needed to trigger disassembly of the virion are measured by applying increasing pressure at one of the three symmetry axes.

AFM has been used to measure the mechanical properties of the virion following integrin αvβ5 or human α-defensin 5 (HD5) binding to the surface of the particle [49]. Association of virions with integrin αvβ5 that interacts with the virus penton base RGD motif, selectively loosened the five-fold (vertex) region of the virus, consistent with the ability of the integrin to promote conformational changes in the capsid vertex region as revealed in cryoEM studies [47]. In parallel AFM studies, binding of α-defensin HD5 to the virus vertex region specifically strengthened the five-fold region of the virus [49]. This finding is consistent with the ability of defensin to stabilize virions in such a way as to block disassembly in the early endosome [5].

AFM nanoindentation experiments have also been used to gain insights into the changes in AdV capsid stability during maturation, a process that may mimic some of the events in capsid disassembly [54,56]. Immature HAdV particles, represented by the *ts1* mutant, exhibit greater elasticity than mature virions [54]. Interestingly, applied forces generated by AFM on individual particles more easily dislodged the penton complex from mature virions than in immature (*ts1*) virions [56]. This suggests that the maturation process prepares virions to undergo capsid disassembly at the vertex region.

The mechanical fatigue resulting from maximum indentation forces causes mature virions to crack open and release their DNA content, which remain tethered to the capsid shell [54,57]. When the resulting DNA-protein cores were analyzed by AFM, it was discovered that *ts1* cores were more rigid than mature cores, suggesting that the maturation process leads to decompaction of the nucleic acid [58]. The modest internal pressure of the inner capsid created by nucleic acid rearrangement during maturation is thought to facilitate capsid disassembly and DNA release once the vertex region becomes dissociated.

## 6. Role of Endosome Acidification in Capsid Disassembly

Both enveloped and nonenveloped viruses utilize environmental cues to initiate conformational changes in the particle to stimulate disassembly. One of the first detailed studies of adenovirus entry by Greber and colleagues showed that inhibitors of low pH (e.g., ammonium chloride) prevented infection during the early phase of cell entry but did not appear to have a role in virus disassembly (i.e., fiber release) [4]. Exposure of virus to low pH buffer also destabilizes virions as measured by the release of the virus vertex region [6]. Unfortunately, chemical modifiers of endosome acidification, such as chloroquine, ammonium chloride, and bafilomycin A1, have significant pleiotropic effects on different cell types, including toxicity that obscures the interpretation of the role of endosome acidification in virus disassembly and cell entry. Moreover, some studies have indicated that chemical inhibitors of low pH do not completely block adenovirus infection even at non-toxic concentrations [59,60] or have no observable effects on cell entry or infection [61,62,63]. Some evidence suggests that virion escape from the endosome, as measured by an indirect method of ribotoxin delivery, requires low pH, as indicated by studies with antiviral cyclic d,l-α-peptides that counteract the development of a low pH environment inside of the endosome [64]. Another study using an assay that quantifies endosomal and cytosolic capsids by Suomalainen et al. [65] indicates that penetration from endosomes does not require low pH. The role of endosomal factors rather than acidification in adenovirus penetration is unknown. It is possible that the proton gradient in the endosome is more important than acidification [66] or that as yet to be identified pH-dependent endosomal proteases could be involved [66].

## 7. Role of the Viral Cysteine Protease (AVP) in Capsid Disassembly

Mature adenovirus particles contain 7–15 copies of a late gene 3 (L3)-encoded 23 kDa cysteine protease (AVP, adenoviral protease) [12,22] that mediates several functions in the virus infectious cycle. One of the most important roles of AVP is to convert preproteins in immature virus capsids—pVI, pIIIa, pVII, pVIII, pX, L52/55K, and pTP—into their mature (cleaved) form by recognizing one of two consensus motifs: (M/I/L)XGX-G and (M/I/L)XGG-X [67]. These AVP-dependent polypeptide cleavages are thought to occur in or near the core of immature particles via a unique mechanism. Specifically, AVP, in association with an 11 amino acid C-terminal fragment of protein VI [68,69], slides along viral DNA with Brownian-like motion and cleaves substrate proteins as it progresses along the nucleic acid “railroad” [70]. The ultimate result of AVP-mediated capsid protein cleavage is the conversion of immature particles into mature virions that can respond to environmental cues (i.e., receptor interactions) to initiate uncoating. This process has also been referred to as capsid priming [71,72]. An alteration of the AVP sequence prevents its incorporation into the capsid during virus assembly in a temperature-sensitive mutant HAdV-C2-*ts1* [12]. Consequently, *ts1* virions contain uncleaved precursor capsid proteins [73,74] and are hyperstable, non-infectious, and incapable of undergoing disassembly in the endosome during cell infection. The underlying causes for the inability of *ts1* particles to undergo disassembly remain to be uncovered. Previous cryoEM structural analyses comparing mature virions with *ts1* particles provided a few clues to this defect [75,76]. These structural studies (at ~10 Å resolution), revealed that *ts1* particles have a different core structure in which there is a more intimate connection with the inner capsid shell. This closer contact could presumably stymie capsid disassembly. However, higher resolution structural studies are needed to reveal the exact protein–protein or protein–DNA associations that restrict virus uncoating.

An outstanding question regarding virus uncoating is whether AVP cleavage of a specific capsid protein plays a crucial role in converting the immature virion to a mature particle that is capable of undergoing disassembly during cell infection. Recently, Moyer and colleagues provided evidence that AVP cleavage of preprotein VI has a significant impact on the ability of immature virions to evolve into mature particles [9]. The N-terminal 33 amino acid residues of preprotein VI mediate hexon association during virus assembly but maturation cleavage by AVP at glycine 32 separates this peptide from the mature protein VI molecules located on the inside of the virus capsid. However, the cleaved 33 residue VI peptide stays associated with the virion during maturation [77] and is visible inside the hexon trimer cavity in the virus crystal structure [2]. The normal AVP-mediated cleavage at the conserved glycine 33 residue in preprotein VI can be abrogated by site-directed mutagenesis, G33A [9]. Importantly, adenovirus mutants containing this G33A mutation in preprotein VI, show significantly reduced infectivity as well as a lower capacity to undergo capsid assembly/maturation, strongly indicating that AVP cleavage at this specific location in the virus plays a substantial role in preparing the viral capsid to undergo transition to a mature virus particle. Further structure/function studies will be necessary to fully understand how the relatively conservative substitution, G33A, in preprotein VI, leads to a significant alteration in virus functions.

## 8. Human α-Defensins Inhibit Adenovirus Infection by Blocking Uncoating

In 2008, Smith and Nemerow showed that human α-defensins, which are small disulfide bonded proteins of the innate immune system, have anti-viral activity against adenovirus [5]. Inhibition of adenovirus was demonstrated with low micromolar concentrations of defensin. This study also showed that direct contact of defensin with the virus was required for neutralization and that defensin stabilizes the capsid and inhibits release of protein VI. A single-cell based assay was developed in order to determine whether or not defensin limits exposure of the viral genome [10]. This study provided direct evidence that human α-defensins block adenovirus uncoating and genome exposure. Both of these studies showed that defensin does not prevent HAdV-C5 from entering host cells [5,10].

Further studies showed that sensitivity of HAdV to defensins is species type specific [78]. Types representative of HAdV species A–F were tested with two different α-defensins: HD5 and human neutrophil peptide 1 (HNP1). Sensitivity to HD5 was found for types of species A, B1, B2, C and E. In general, those types that are sensitive to HD5 are also sensitive to HNP1. One exception is that HAdV-E4 is moderately sensitive to HD5 and resistant to HNP1. Notably, HAdV types from species D and F were resistant to both α-defensins. These results indicate that there is a sequence-dependence to defensin sensitivity. The same study showed that the interaction between HAdV-C5 and HD5 can reach saturation. At the saturation point, approximately 2750 HD5 molecules are bound per virion. When a form of HD5 without its normal three disulfide bonds (HD5-Abu) was tested for viral inhibition, it was found to be ineffective in neutralization assays. This indicates that the tertiary structure of HD5 is important for neutralization, not just its net positive charge, which is preserved for HD5-Abu.

Several cryoEM structures of adenovirus α-defensin complexes have been determined [78,79]. The first of these studies was with a HAdV vector with a short fiber, called Ad5.F35 or Ad35F [78]. The moderate (~12 Å) resolution the Ad5.F35-defensin complex structure was compared to a previous Ad5.F35 cryoEM structure at ~7 Å resolution [1]. Density attributed to defensin was found on top of the penton base and surrounding the fiber shaft, as well as on the flexible loops at the top of hexon. In order to determine which of the many HD5 binding sites might be the critical site for neutralization, the authors performed an exhaustive sequence alignment of the HAdV capsid proteins. They reasoned that they might find a negatively charged region of the capsid that was present within the HD5 sensitive types and absent within the HD5 resistant types and positioned in the vicinity of cryoEM density attributed to HD5. One such candidate site emerged, in the N-terminal region of the fiber, just after the short peptide region involved in interaction of the fiber with the penton base. In sensitive HAdV types, the fiber sequence at this region is negatively charged (for example DTET in HAdV-C5 or EDES in HAdV-B35). In contrast, the fiber sequence at this site in α-defensin resistant types is positive and hydrophobic (for example GYAR in HAdV-D19c). Density attributed to HD5 was observed at this site on top of the penton base in the Ad5.F35-defensin cryoEM structure.

In order to test the importance of this region of the fiber for α-defensin neutralization, several chimeric vectors were produced [78]. It was found that by making two swaps between a sensitive and a resistant HAdV serotype, it is possible to convert an α-defensin sensitive vector into an α-defensin resistant vector. These two swaps correspond to (1) the identified four residues near the N-terminus of fiber; and (2) the entire penton base protein. These results led to the hypothesis that the HD5 neutralization determinants are located in a region of the capsid spanning the fiber and the penton base.

**Figure 3:**
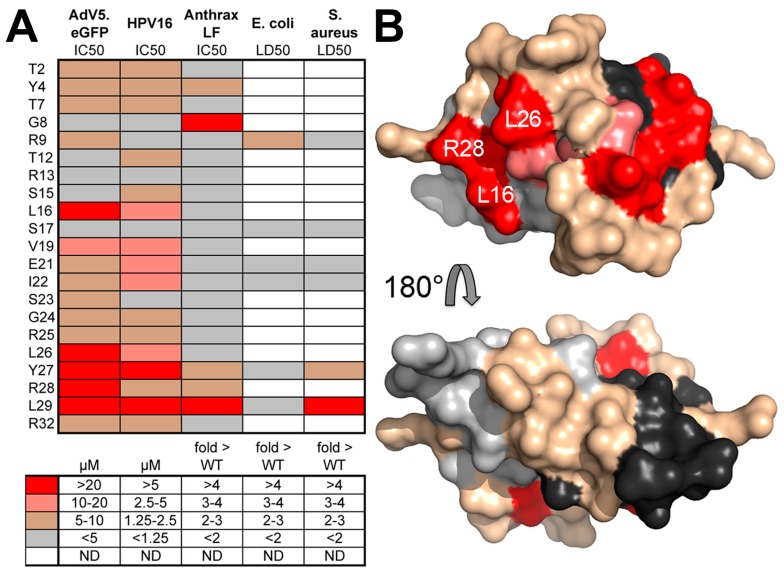
Human α-defensin HD5 residues critical for adenovirus neutralization. (**A**) Heat map of the effect of alanine substitutions on the IC50 of HD5 against a variant of HAdV-C5 (AdV5.eGFP), human papillomavirus 16 (HPV16), and anthrax lethal factor (LF), or on the LD50 of HD5 against *E. coli* and *S. aureus* according to the color key below; (**B**) Two views (front and back) of the HD5 dimer shown in a surface rendering. Residues are colored according to the heat map data in panel A for AdV5.eGFP. Reprinted with permission from [81].

**Figure 4:**
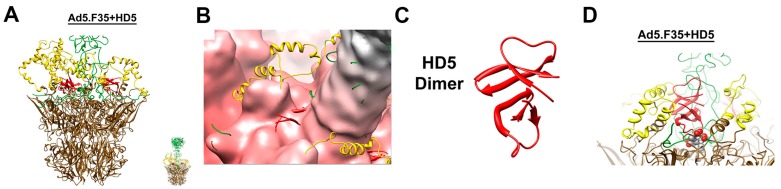
CryoEM and molecular dynamics flexible fitting (MDFF) analysis of the interaction between human α-defensin HD5 and the penton base of a defensin-sensitive adenovirus vector. (**A**) Atomic model of the penton base (brown with flexible RGD loops in yellow) and fiber (green) of Ad5.F35 with HD5 monomers (red) docked at the critical binding sites at the interface between the penton base and fiber. The inset shows the atomic model with the full length fiber; (**B**) Atomic model of the vertex region with HD5 monomers shown docked within the cryoEM density of the complex. Note that cryoEM density is not observed for the flexible RGD loops; (**C**) Ribbon representation of the HD5 dimer (PDB-ID 1ZMP); (**D**) Final MDFF coordinates for one vertex simulation of the Ad5.F35+HD5 interaction shown in side view. The HD5 dimer (red) is in close proximity to the fiber sequence 18-EDES-21 (spheres) and flanked by an RGD-containing loop of the penton base (yellow). Reprinted with permission from [79].

Later studies demonstrated the importance of dimerization and multimerization of HD5 for neutralization of HAdV [80] and probed the surface of the HD5 dimer important for HAdV neutralization [81]. The HD5 residues that are most critical for neutralization of adenovirus were found to be located on one side of the HD5 dimer, and these include Leu-16, Leu-26, and Arg-28 (Figure 3). The critical HD5 binding site on the HAdV capsid was further investigated by cryoEM structural studies of HD5 in complex with both a sensitive and a resistant HAdV vector coupled with molecular dynamics with flexible fitting (MDFF) analysis [79]. The MDFF software package offers a way to perform full-atomistic molecular dynamics simulations with an additional steering force provided by the cryoEM density [82,83]. The cryoEM and MDFF studies indicated that intrinsically disordered regions at the top of the penton base may be involved in the HAdV/HD5 interaction (Figure 4) and that binding of HD5 may stabilize the HAdV vertex region composed of penton base and fiber by a three-fold increase in intermolecular nonbonded interactions [79]. The finding that α-defensin binding may stabilize the vertex region of sensitive HAdV types is consistent with the AFM observation that human α-defensin increases the mechanical strength of the vertex region by almost 70% [49].

## 9. Evasion of Antibody Neutralization

Antibodies elicited against the major outer HAdV capsid proteins such as the hexon, penton base and fiber during adenovirus infections have long been recognized for their ability to mediate virus neutralization [24,84,85,86]. The modes of neutralization range from blocking cell receptor interactions to inhibition of virus capsid uncoating or particle trafficking in cells. Neutralizing antibodies are able to protect the host against disseminated infection as well as re-infection by the same virus type. Unfortunately, pre-existing neutralizing antibodies also serve as a barrier for the use of replication-defective HAdV-C5 based vectors, particularly for systemic administration. Current efforts, particularly in the area of HAdV-vaccine development, are focusing on the use of so-called unusual HAdV types such as HAdV-D26 and HAdV-B35 as well as simian adenoviruses given that pre-existing neutralizing human antibodies for these virus types are not as prevalent as those to HAdV-C5.

Anti-adenoviral antibodies, particularly monoclonal antibodies, have provided unique insights into the mechanisms of neutralization, virus uncoating and intracellular virus trafficking. A cryoEM structural analysis of a monoclonal antibody, designated DAV-1, directed against the internalization RGD motif on the penton base indicated that the IgG molecule is incapable of neutralizing the virus due to steric hindrance from the neighboring fiber protein [45]. In contrast, the relatively smaller Fab fragments derived from the DAV-1 monoclonal antibody were able to neutralize the virus by blocking integrin-mediated uptake into cells. This finding illustrated the “cleverly” designed architecture of the virus in which crucial cell entry motifs on the penton base avoid antibody neutralization while still being accessible for cell receptor-mediated virus uptake.

## 10. Transit of HAdV to the Nuclear Pore Complex and the Final Steps of Uncoating

Adenovirus capsids that manage to avoid neutralization by soluble host factors such as antibodies and defensins still need to navigate their way toward the cell nucleus. Early during infection, adenovirus capsids that have escaped from the early endosomes remain partially uncoated after shedding the fiber, penton base and protein VI proteins. These changes in the virion are thought to facilitate dynein motor binding to the capsid [16]. Hypervariable region 1 (HVR1) appears to be the prime candidate for the dynein binding site on the hexon [16] while the region of the dynein motor that mediates this association is still uncertain. Phosphorylation of the dynein light chain is thought to be necessary for virus binding [87]. In addition to the role of the hexon in directing the virus to the nucleus, protein VI has been suggested to facilitate microtubule transport via an PPXY motif that presumably is exposed upon partial capsid disassembly [88]. However, this process may be limited by the fact that capsid associated protein VI is rapidly degraded early during cell infection [89].

Newly emerging information indicates that transport of the virus to the nuclear pore occurs via microtubules and dynein motors [90,91,92,93]. The final “undressing” of the adenovirus capsid at the nuclear pore complex is necessitated by the observation that macromolecular complexes of greater than 40 nm in diameter are unable to enter the nucleus by simple passive diffusion [94]. Host molecules such as karyopherins (importin α) and RAs-related Nuclear protein-Guanosine-5'-triphosphate (Ran-GTP) recognize potential nuclear cargo and assist entry of large, complex biomolecules such as viral double stranded DNA (dsDNA) and associated core proteins. HAdV capsid docking to the nuclear pore complex appears to be mediated by the hexon association with a specific nucleoporin, Nup214 on the outside of the nuclear pore complex [95,96]. The action of kinesin-1 along with nucleoporin associations have been purposed to provide the mechanical forces required for the disruption of the capsid, thereby allowing the viral genome to enter the nucleus [19], likely aided by nuclear localization motifs present on protein VII that is tightly bound to the nucleic acid. The details of the final step in adenovirus uncoating remain to be elucidated but these recent studies add another chapter to our understanding of the very close linkage between capsid disassembly and nuclear transport in the virus cell entry pathway.

## 11. Summary and Future Studies

Adenoviral cell entry begins with the interaction of the virus with two host cell receptors, typically CAR and αv-integrins. These initial two viral/host interactions trigger fiber release at the surface of the host cell, which is the first step in adenovirus uncoating. Viral interaction with integrins leads to further conformational changes and weakening of the capsid vertex. Release of the internally packaged membrane lytic protein VI is a necessary step for escape of adenovirus from the endosome. The exact triggers for further adenovirus disassembly within the endosome remain unclear. Following endosomal escape, transport of the partially uncoated adenovirus virions to the nuclear pore complex must take place for delivery of the viral genome to the nucleus of the host cell.

Human adaptive and innate immune molecules are now well recognized for their ability to block the earliest steps in cell attachment, internalization or intracellular trafficking in many different virus systems. Analyses of these immune-mediated neutralization steps with human adenovirus continue to shed important light on the processes involved in cell entry and adenovirus capsid disassembly. The discovery that certain α-defensins block adenovirus uncoating in the early endosome was unexpected and illustrates the diverse mechanisms by which host molecules operate in defense of the host.

These findings also raise the question as to whether there might be as yet unidentified host defense restriction factors that block adenovirus association with the nuclear pore complex or the final disassembly of the capsid at the nuclear pore complex, or perhaps interfere with viral DNA import into the nucleus. Although such neutralization processes have not yet been identified with adenovirus, other viruses such as herpes simplex virus I [97] and influenza viruses [98,99] are susceptible to restriction by host factors that block viral nucleic acid transport or residence in the nucleus. Such discoveries have added new knowledge on the role of host cell responses to virus infection. They also have the potential of spawning the development of novel antiviral agents that selectively target virus uncoating or nuclear transport [100,101].

It is clear that the interaction of adenovirus with host cells is complex and that productive infection requires completion of an intricate series of disassembly steps. There is the potential that understanding the adenoviral disassembly/assembly pathways in detail will lead to the design of new antivirals targeting these pathways. Certainly, as we learn more about adenoviral cell entry, we will continue to gain further insights into normal host cell processes and defense mechanisms.
